# Preparation of Cotton–Zinc Composites by Magnetron Sputtering Metallization and Evaluation of their Antimicrobial Properties and Cytotoxicity

**DOI:** 10.3390/ma15082746

**Published:** 2022-04-08

**Authors:** Marcin Henryk Kudzin, Małgorzata Giełdowska, Paulina Król, Zuzanna Sobańska

**Affiliations:** 1 Łukasiewicz Research Network—Łódz Institute of Technology, 19/27 Marii Sklodowskiej-Curie Str., 90-570 Łódz, Poland; malgorzata.gieldowska@lit.lukasiewicz.gov.pl (M.G.); paulina.krol@lit.lukasiewicz.gov.pl (P.K.); 2 Nofer Institute of Occupational Medicine, Św. Teresy od Dzieciątka Jezus 8, 91-348 Łódz, Poland; zuzanna.sobanska@imp.lodz.pl

**Keywords:** antimicrobial activity, coating, composite, cotton, cytotoxicity, magnetron sputtering, zinc

## Abstract

The aim of this investigation was to evaluate the biological properties of cotton–zinc composites. A coating of zinc (Zn) on a cotton fabric was successfully obtained by a DC magnetron sputtering system using a metallic Zn target (99.9%). The new composite was characterized using scanning electron microscopy/energy-dispersive X-ray spectroscopy (SEM/EDS), UV/Vis transmittance, and atomic absorption spectrometry with flame excitation (FAAS). The composite was tested for microbial activity against colonies of Gram-positive (*Staphylococcus aureus*) and Gram-negative (*Escherichia coli*) bacteria and antifungal activity against *Aspergillus niger* and *Chaetomium globosum* fungal mold species as model microorganisms. Cytotoxicity screening of the tested modified material was carried out on BALB/3T3 clone mouse fibroblasts. The SEM/EDS and FAAS tests showed good uniformity of zinc content on a large surface of the composite. The conducted research showed the possibility of using the magnetron sputtering technique as a zero-waste method for producing antimicrobial textile composites.

## 1. Introduction

Cotton is a popular fabric based on cellulose [1,2]—the most widespread biopolymer on Earth [3,4,5]. Consequently, the physicochemical properties of a cotton fabric follow from the cellulose polymer and depend on its chain length, topology, and surface condition [6,7,8,9,10]. One of the many applications of cellulose is as medical textiles, mainly wound dressings with various functions and purposes. However, the hydrophilic nature of cotton and its products, due to their large specific surface area and hydrophilicity, provide an excellent environment for the development of pathogenic microorganisms [11,12,13]. Therefore, antibacterial pre-functionalization of cotton intended for medical use is a standard finishing step, usually based on equipping the cotton matrix with organic/inorganic antimicrobials [12,14,15,16].

The wide group of these antimicrobial agents can be divided into organic (antibiotics, e.g., [17], quaternary ammonium salts [18,19,20], light-activated singlet oxygen generators [21,22], *N*-halamines [23]), and inorganic factors (halogens as well as heavy metals and their salts) [12,16]. Among various inorganic bactericides of medical importance, zinc is gaining more and more attention (over 5600 documents on the antibacterial activity of zinc and compounds and over 3000 on zinc oxide abstracted by Scopus) [24,25]. Zinc is indispensable in many biological processes [26], toxic to microorganisms, and nontoxic to higher organisms [27]. This chemical element is a cheap and effective antibacterial inorganic compound [14,28], due to its low cost and easy preparation [29,30,31,32,33,34,35,36,37,38], as well as antimicrobial efficacy [29,39,40,41,42,43,44]. The chemistry of zinc is dominated by the oxidation state of (+2). Metallic zinc is a strong reducing agent (standard reduction potential E_0_ = −0.76 V), reacting readily with acids, alkalis, and other nonmetals. The metal surface reacts with atmospheric components, thus eventually creating a protective passivating layer of basic zinc carbonate, xZn(OH)_2_/ZnOxyZnCO_3_ [45,46]. TEM studies of Zn nanoparticles (ZnNPs) showed that ZnNPs consist of a zinc core and a few nanometer thick ZnO shell [47]. Bulk oxidation of zinc in air takes place at temperatures above 450 °C [48]. The standard molar enthalpies of ZnO formation (ΔfH° solid (298.15 K·(kJ·mol^−1^)) were found to be dependent on the sample morphology, varying from −350.46 for bulk ZnO [49] to −336.57 and −343.56 for various ZnO nanoparticles [50,51]. The properties and applications of ZnO were reviewed by Ellmer and Klein [52]. Both Zn and ZnO are common target materials for magnetron sputtering-coated fabrics [53]. Strong acids, such as hydrochloric acid, remove the passivating layer, and the subsequent reaction with acid releases hydrogen gas. The predominant species in aqueous solution is the octahedral complex (Zn(H_2_O)_6_)^2+^ [45]. The bactericidal mechanism of metal/metal oxide nanocomposites (Zn is always coated with ZnO as a result of surface passivation) consists of the production of reactive oxygen species (superoxide anions, hydrogen peroxide anions, and hydrogen peroxide) that interact with the bacterial cell wall causing damage to the cell membrane and then leakage of internal cellular components, leading to the death of bacteria [54,55,56,57]. Alternatively, ZnO-NCs in contact with bacteria release Zn^2+^ ions [29,58] which penetrate the cell membrane, destroying its normal structure and function, and consequently causing the death of bacterial organisms [59,60]. The bactericidal inactivation by ZnO performed under dark/light conditions is attributed to the bactericidal effect of Zn^2+^ ions under dark conditions or to the combined effects of Zn^2+^ ions and photocatalytically mediated electron injection under light conditions [61]. Due to the antibacterial properties of zinc oxide, several cotton–zinc oxide composites (COT–ZnO) have been proposed for medical applications [62,63,64,65,66,67,68,69,70,71,72,73]. Representative examples of antibacterial cotton using zinc salts are shown in Table 1.

The modification of textiles by means of magnetron sputtering does not require the use of any chemicals, can be achieved in one process cycle in a single industrial installation, and does not involve the emission of toxic substances to the environment or the production of pollutants [53,74,75,76,77,78,79,80]. Therefore, this method can be considered eco-friendly and zero-waste.

Zinc magnetron sputtering is one of the techniques frequently used in modern science and technology (11,739 documents on zinc sputtering abstracted by Scopus [81]), as well as in relation to sputtering of zinc on polymers (283 documents on polymer zinc sputtering [82]). Thus, zinc oxide (ZnO) films have been deposited on polyethylene terephthalate (PET) [83,84,85,86,87,88,89,90,91], polyethylene naphthalate (PEN) [79,83,92,93], polytetrafluoroethylene (PTFE) [94,95,96,97], and poly(acrylic acid) [98], as well as on the surface of polypropylene (PP) fibers [99], polystyrene [100], and poly(ether ether ketone) (PEEK) films [101].

Only a few studies have been devoted to other zinc compounds such as zinc sulfide (ZnS) deposited on polyethylene terephthalate (PET) [102] and gallium-doped zinc oxide (GZO) deposited on a transparent flexible substrate based on cellulose derivatives [103]. Only in two studies was metallic zinc used for surface functionalization of polymer nanofibers, namely, for polyamide [104], and for polyethylene (PE) and polytetrafluoroethylene (PTFE) [105].

As part of our research program dedicated to biologically active functionalized phosphonates [106,107] and biofunctionalization of textile materials [108,109,110,111,112,113,114], we present the preparation and physicochemical and biological properties of the COT–Zn polymer hybrid. The aim of this work was to modify the surface of cotton fabric with zinc using the DC (direct current) magnetron sputtering method to produce a new antimicrobial, multifunctional composite material.

## 2. Materials and Methods

### 2.1. Materials

#### 2.1.1. Composite Components

- Medical fabric with a plain weave; qualitative composition: cotton (100% *w*/*w*); weight: 200 g/m^2^ (Andropol S.A., Andrychów, Poland);
- Zinc sputtering target with 99.9% purity with a rectangular size (798 × 122 × 6 mm) (Testbourne Ltd., Basingstoke, UK).

#### 2.1.2. Bacterial and Fungal Strains

The following bacterial strains and fungal strains were purchased from Microbiologics (St. Cloud, MN, USA):

- *Escherichia coli* (ATCC 25,922, Microbiologics, St. Cloud, MN, USA);
- *Staphylococcus aureus* (ATCC 6538, Microbiologics, St. Cloud, MN, USA);
- *Aspergillus niger* (ATCC 6275, Microbiologics, St. Cloud, MN, USA);
- *Chaetomium globosum* (ATCC 6205, Microbiologics, St. Cloud, MN, USA).

#### 2.1.3. Cell Culture

BALB/3T3 clone A31 cell line cat. no. CCL-163 (mouse fibroblasts) was purchased from the American Type Culture Collection/the Global Bioresource Center (ATCC, Manassas, VA, USA).

### 2.2. Methods

#### 2.2.1. Magnetron Sputtering

The medical cotton fabric (COT) was modified using a DC (Direct Current) magnetron sputtering system produced by P.P.H. Jolex s.c. (Czestochowa, Poland) and a zinc target. The distance between the target and the substrate was 15 cm. The deposition of coatings was carried out in the atmosphere of argon. The following parameters were used for the modification: discharge power 700 W, with the resulting power density 0.72 W/cm^2^ and working pressure 2.0 × 10^−3^ mbar. In order to differentiate the zinc content (Zn^(0)^ + Zn^(2+)^) in the composites COT–Zn, three different deposition variants were used, i.e., 5 min one-sided (sample name: COT–Zn-5(1 s)), 10 min one-sided (COT–Zn-10(1 s)), and 10 min two-sided (COT–Zn-10(2 s)).

#### 2.2.2. SEM/EDS—Scanning Electron Microscopy/Energy-Dispersive X-ray Spectroscopy

The microscopic structure was examined using a HITACHI S-4700 scanning electron microscope (Tokyo, Japan) equipped with a Thermo NORAN EDS X-ray microanalyzer (Waltham, MA, USA). The topography analysis of the tested samples was carried out in low vacuum at a beam energy of 10 kV and magnifications of 400×, 1600×, and 3000×. The study was conducted under low vacuum in the presence of steam. Water vapor dissipates excess charge, making it possible to image nonconductive materials without coating the surface with gold.

#### 2.2.3. Atomic Absorption Spectrometry with Flame Excitation—FAAS

Zinc content in the composite samples was determined using the Thermo Scientific Thermo Solar M6 atomic absorption spectrometer (Waltham, MA, USA) equipped with a 100 mm titanium burner, coded lamps with a single-element hollow cathode, and a D2 deuterium lamp for background correction. The sample was prepared using a single-module Magnum II microwave mineralizer from Ertec (Wroclaw, Poland).

The total zinc content of the sample *M* (mg/kg) was calculated according to the following formula [115]:

(1) $$M=\frac{C_{i}\times V}{m}\;\left[\frac{\mathrm{mg}}{\mathrm{kg}}\right],$$

where *C* is the Zn concentration in the tested solution (mg/L), *m* is the mass of the mineralized sample (g), and *V* is the volume of the sample solution (mL).

#### 2.2.4. Biological Experiments

##### Antibacterial Activity

The antibacterial activity of COT–Zn composites was tested by the agar (Mueller Hinton medium) diffusion method (PN-EN ISO 20,645:2006), on colonies of Gram-negative (*E.* *coli*; ATCC 25,922) and Gram-positive (*S. aureus*; ATCC 6538) bacteria [116]. The test was initiated by pouring each agar into sterilized Petri dishes and allowing it to solidify. The surfaces of agar media were inoculated with the overnight broth cultures of bacteria (ATCC 25,922: 1.5 × 10^8^ CFU/mL, ATCC 6538: 2.5 × 10^8^ CFU/mL). Samples of sterile COT–Zn discs and a control, unmodified sample (10 mm) were placed on the inoculated agar and incubated at 38 °C for 24 h. The diameter of a clear zone around the sample was measured as an indication of inhibition of the microbial species. All tests were carried out in duplicate.

##### Antifungal Activity

The antifungal activity of the COT–Zn composites was tested according to PN–EN 14,119:2005 against *A. niger* (ATCC 6275) and *C. globosum* (ATCC 6205) [117]. Specimens of the tested material were placed on agar plates; the samples of sterile modified COT–Zn discs (20 mm) and the control, unmodified sample were placed on inoculated agar (pH:6.2) and incubated at 30 °C for 14 days. The agar was inoculated with the selected fungus (ATCC 6275: CFU/mL = 3.5 × 10^6^, ATCC 6205: CFU/mL = 3.0 × 10^6^). The level of antifungal activity was assessed by examining the extent of fungal growth: in the contact zone between the agar and the specimen, on the surface of specimens, and, if present, the extent of the inhibition zone around the specimen. All tests were carried out in duplicate.

#### 2.2.5. Cytotoxicity

##### Cell Culture

Cells (mouse fibroblasts: BALB/3T3 clone A31 cell line cat. no. CCL-163) were grown in T-25 culture flasks in a humidified CO_2_ incubator (37 °C, 5% CO_2_) and maintained in a culture medium (cDMEM), i.e., DMEM medium (Biowest, Riverside, CA, USA) with 10% fetal bovine serum (Gibco, Waltham, MA, USA), 100 U/mL penicillin, 100 µg/mL streptomycin (Biowest, Riverside, CA, USA), 4 mM l-glutamine (Biowest, Riverside, CA, USA), and 20 mM HEPES (Sigma-Aldrich, Saint Louis, MO, USA). Cells were examined daily using an Olympus IX70 inverted microscope (Tokyo, Japan) and were routinely passaged twice a week when reaching 60–70% confluency. Next, they were detached using 0.25% trypsin-EDTA solution (BI, Kibbutz Beit-Haemek, Israel) (5 min, 37 °C) and seeded in 96-well plates (3.5 × 10^3^ cells/well). Then, the cells were allowed to adhere for 22–24 h in the CO_2_ incubator.

##### Extract Preparation and Cell Treatment

Extracts of test materials (COT and COT–Zn composites) were prepared according to EN ISO 10993-12-2012 [118] using the exposure medium, i.e., cDMEM with a lower (5%) concentration of FBS, in order to avoid the masking of toxicity by the protective effect of the serum. Test materials were autoclaved (120 °C, 20 min) and left at 70 °C for 5 h to dry. Next, the materials were immersed in the exposure medium using the extraction ratio of 0.1 g of the material to 1 mL of the exposure medium, i.e., the predetermined additional volume of the exposure medium needed for the maximum soaking of the test material. Extraction was performed in shaken vials that were incubated at 37 °C for 24 h. The pH values of extracts (ca. 8) were adjusted to 7.4 using 1 N HCl (POCH, Gliwice, Polska). Extracts, the preparations, and sample abbreviations are summarized in Table 2.

Cells were exposed to the extracts at selected concentrations (100% and 50% for unmodified cotton; 100%, 50%, and 25% for modified cotton) for 24 h, i.e., after 24 h adherence of cells, the supernatant above the cells was aspirated and replaced with 100 µL of an appropriately concentrated extract or control solution (negative control, NC—exposure medium treated in the same way as extracts; positive control—SDS (Sigma-Aldrich, Saint Louis, MO, USA) in the concentration range 0–150 µg/mL). During the experiment cells were examined with the Olympus IX70 (Tokyo, Japan) inverted microscope. Each sample was tested in triplicate per experiment, and three independent experiments were performed.

##### The Neutral Red Uptake (NRU) Assay

After the 24 h incubation of BALB/3T3 clone A31 cells with the extracts, the medium was gently aspirated, and the wells were washed with 150 µL of Dulbecco’s phosphate-buffered saline (PBS) with Ca^2+^ and Mg^2+^ ions (BI, Kibbutz Beit-Haemek, Israel). Then, 100 µL of NR (Sigma-Aldrich, Saint Louis, MO, USA) solution (50 µg/mL, prepared in culture medium) was added to each well, and the plates were incubated further for 3 h (37 °C; 5% CO_2_). Afterward, the NR solution was removed, the wells were washed with 150 µL of PBS, and 150 µL of desorbing solution [1% glacial acetic acid (POCH, Gliwice, Polska), 50% ethanol (POCH, Gliwice, Polska), and 49% deionized water] was added to each well. Absorbance was read at 540 nm using a Multiskan™ GO spectrophotometer (Thermo Fisher Scientific, Waltham, MA, USA). Results were expressed as the percentage cell survival (OD of exposed vs. OD of control unexposed cells).

## 3. Results and Discussion

### 3.1. SEM/EDS—Scanning Electron Microscopy/Energy-Dispersive X-ray Spectroscopy

**Figure 1:**
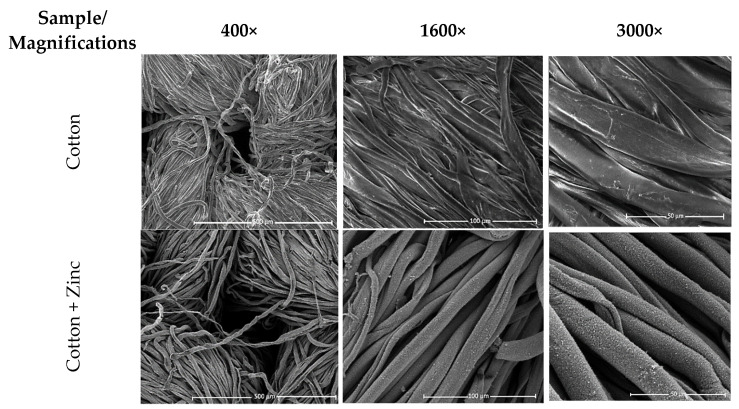
SEM results (magnifications: 400×, 1600×, and 3000×) of the tested samples recorded before and after magnetron sputtering with a zinc target.

The analysis of changes in the morphological structure of fibers in cotton fabric under zinc modification was carried out using scanning electron microscopy. Figure 1 shows SEM images of the samples before and after magnetron sputtering, at magnifications of 400×, 1600×, and 3000×.

According to the image analysis, it can be noted that the fibers in cotton fabric were characterized by a rather smooth surface with characteristic parallel ridges and grooves. The fibers in cotton fabric with a magnetron-sputtered zinc layer were characterized by a rougher surface with visible ridges and grooves in the fibers. The zinc coating exhibited a regular distribution of the applied modifier particles.

**Figure 2:**
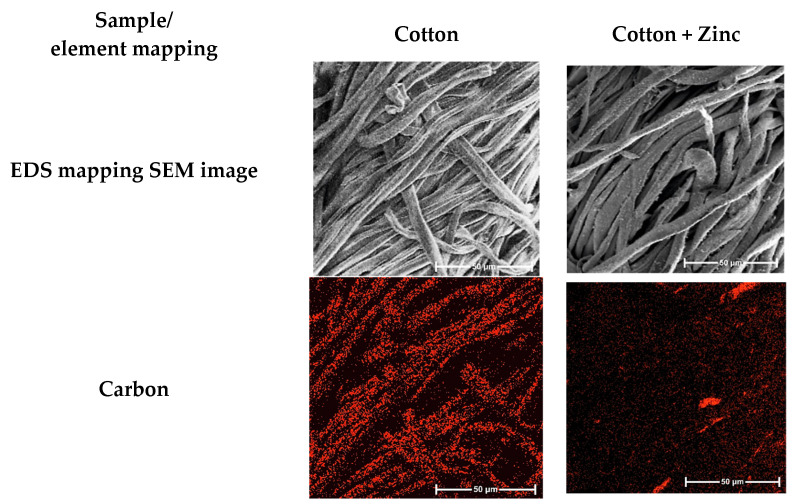

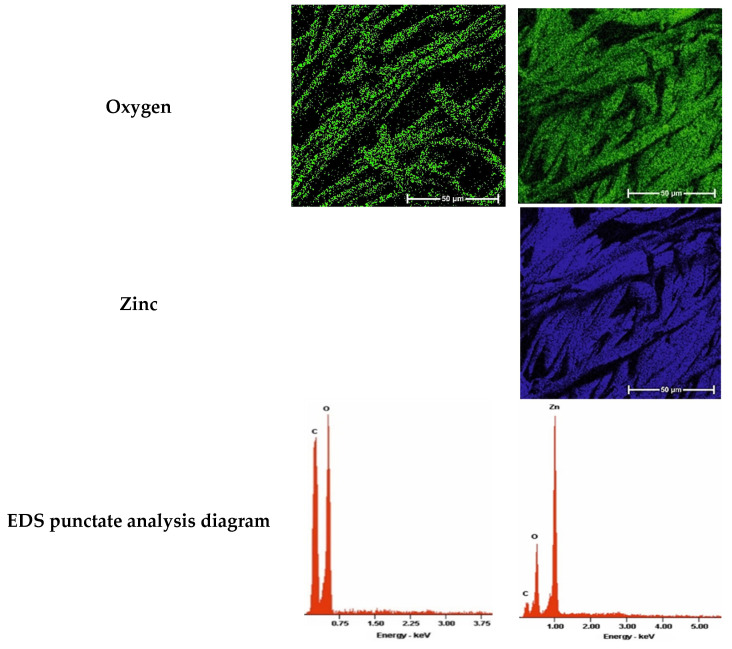
Multi-element EDS mapping images and EDS spot analysis diagram: analysis of the chemical composition of COT and COT–Zn sample.

In order to confirm and verify the uniformity of the zinc coating of the fibers, an energy-dispersive X-ray spectroscopy (EDS) study was performed, which provided a chemical analysis of the tested fabric and its elemental composition. The EDS surface analysis (Figure 2) in the form of individual “mapping” and the quantitative spot analysis showed the content of characteristic elements in the cotton fiber surface.

The study indicated a uniform distribution of zinc on the surface-modified fibers. The black areas visible in the images were the spaces between the fibers in the yarn (three-dimensional structure). Due to the dense and uniform zinc coating of the fibers, carbon became less visible, which was confirmed by the quantitative analysis of the content of elements on the fiber surface (Table 3) and EDS punctate analysis diagram (Figure 2).

The nature of the so-called zinc–cellulose interface matter (Zn–cell–OH) still remains unclear, since the zinc atom is reactive, and cellulose possesses reactive hydroxyl functions. It is well documented that zinc reacts with alcohols with the temporary formation of zinc alcoholate intermediates, which, in an aqueous environment, rearrange into zinc oxide [119,120,121,122].

**Figure 3:**
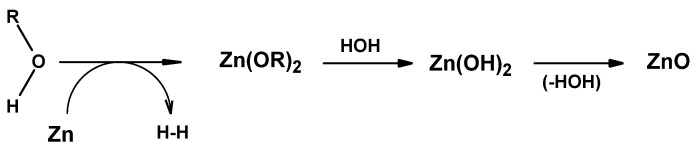
Putative mechanism of the reaction of zinc with alcohols (R = Me or Et) [119,120,121,122].

The reactions of zinc with alcohols presumably proceeded in accordance with Figure 3.

**Figure 4:**
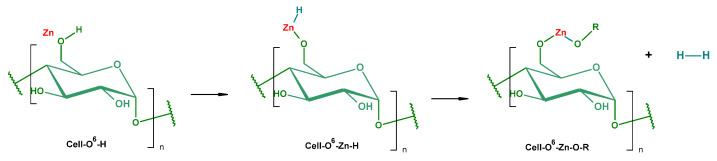
Putative mechanism of the reaction of zinc with cellulose during sputtering deposition of zinc on cotton.

Subsequently, zinc atoms of a metallic monolayer deposited on the cellulose surface reacted during deposition with cellulose, forming corresponding alcoholates (Figure 4), which could subsequent hydrolyze to cellulose and ZnO.

The following layers of deposited zinc atoms were attached to the lower zinc layer, forming appropriate zinc multilayers. The zinc atoms of the upper layer were oxidized to ZnO, by reaction of zinc atoms with oxygen or water. This mechanism was partly confirmed by EDS test (Table 3). The results for COT were as follows: C (40.0%), O (60.0%); the results for COT–Zn-10 (1 s) were as follows: C (3.2%), O (17.7%), Zn (79.7%). This suggests that carbons of the cellulose skeleton were covered by O–Zn moieties. Cellulose-derived oxygens were masked by zinc atoms; hence, the oxygen revealed in the EDS test was presumably derived from the passivated layer, i.e., from ZnO. The ratio of Zn:O ≈ 4.5:1 (Zn_1.2_O_1.1_) suggests a nearly quantitative character of passivation. It is worth adding that the formation of ZnO during sputtering with pure zinc has been described in a few papers using pure Zn target in an argon–oxygen [123,124,125] or oxygen atmosphere [126]. Another example of the formation of a metal–polymer interface during Zn sputtering onto PE, PTFE, and PI surfaces was described by Pertsin and Volkov [105].

### 3.2. Atomic Absorption Spectrometry with Flame Excitation—FAAS

The determination of zinc content in COT–Zn composite samples was assessed by the FAAS method, and the results are presented in Table 4.

The zinc content in composite samples strictly depended on the applied sputtering metallization times. The process of Zn deposition on the cotton sample was practically linear (COT–Zn-5 (1 s)—9.06 g/kg, COT–Zn-10 (1 s)—20.19 g/kg, COT–Zn-10 (2 s)—41.52 g/kg), and the distribution of metal in COT–Zn^(0)^ material bulk after modification was uniform.

### 3.3. Antimicrobial Properties

#### 3.3.1. Antibacterial Activity

**Figure 5:**
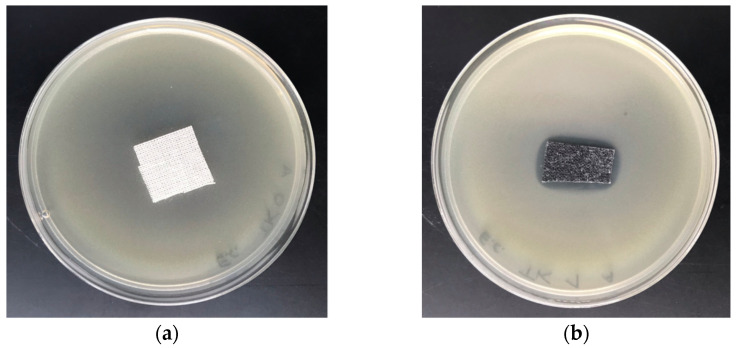
Tests of antimicrobial activity of COT–Zn^(0)^ composites against *E. coli*. Inhibition zones of bacterial growth in Petri dishes: (**a**) COT; (**b**) COT–Zn-10 (1 s/2 s).

**Figure 6:**
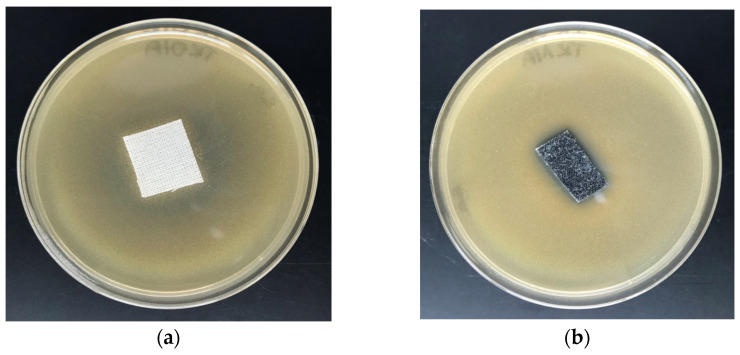
Tests of antimicrobial activity of COT–Zn^(0)^ composites against *S. aureus*. Inhibition zones of bacterial growth in Petri dishes: (**a**) COT; (**b**) COT–Zn-10 (1 s/2 s).

The COT–Zn composites were tested in vitro for antimicrobial activity against Gram-positive *S. aureus* and Gram-negative *E. coli*. Results of the tests are illustrated in Figure 5 and Figure 6.

A comparison of these results (ZID) with representative data from the literature is given in Table 5.

The literature data related to the antibacterial activity of zinc-based composites cannot be used as direct comparative data, due to the difference in the applied test methods. The presented results (Table 5) indicate that, independently of the type of base material used for modification with Zn and zinc compounds, and irrespective of the applied test method to evaluate antimicrobial activity, the expected result was achieved. Results of these tests demonstrate the antimicrobial protection against various bacterial microorganisms of COT–Zn-10 (1 s/2 s) composites for *E.* *coli* and *S.* *aureus* (Table 5), expressed by the visible zones of inhibition of bacterial growth on the Petri dishes (Figure 5b and Figure 6b).

#### 3.3.2. Antifungal Activity

**Figure 7:**
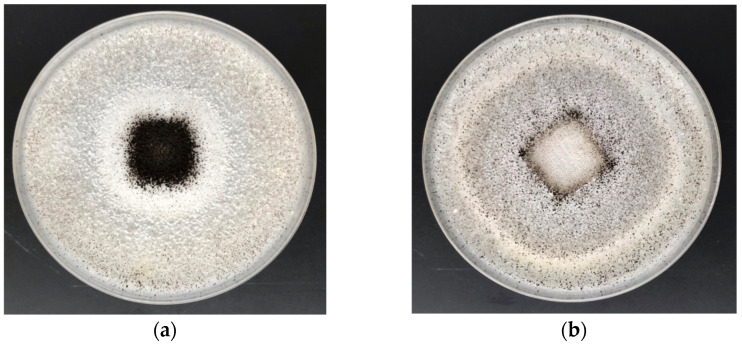
Antimicrobial activity tests against *A. niger*. Inhibition properties of fungal growth in Petri dishes: (**a**) COT; (**b**) COT–Zn-10 (1 s/2 s).

**Figure 8:**
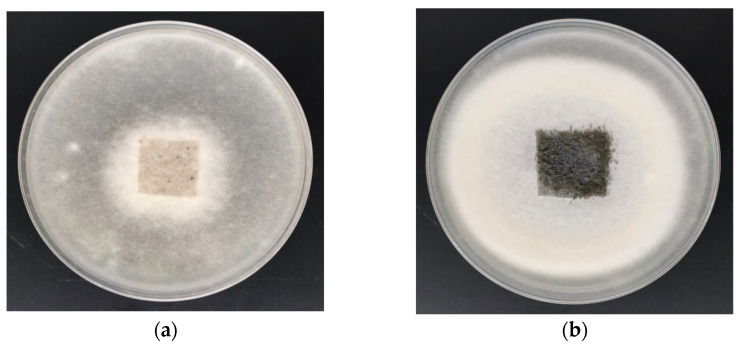
Antimicrobial activity tests against *C. globosum*. Inhibition properties of fungal growth in Petri dishes: (**a**) COT; (**b**) COT–Zn-10 (1 s/2 s).

The results of the antifungal activity tests (ZID) in accordance with PN-EN 14119:2005 against colonies of *A. niger* (ATCC 6275) and *C. globosum* (ATCC 6205) for the cotton sample and COT–Zn composites are illustrated in Figure 7 and Figure 8 and presented in Table 6 (comparison with representative data from the literature).

As anticipated, the unmodified sample (100% cotton) did not inhibit the growth of *A. niger* or *C. globosum*, as expressed by the strong visible fungal growth covering the entire surface of the COT samples (Figure 7a and Figure 8a). Antifungal activity and protection against *A. niger* and *C. globosum* were demonstrated by COT–Zn-10 (1 s/2 s) samples modified by magnetron sputtering metallization. The results revealed visible zones of fungal growth inhibition in Petri dishes (Figure 7b and Figure 8b), with no fungal growth on the surface of the composites.

The zinc oxide impact on bacteria or fungi depends on its morphology (particle size and shape), concentration, exposure time, pH, etc. [148]. This is illustrated by the corresponding ZID values summarized in Table 5 and Table 6. Generally, ZnO NPs had ZID values over 10 mm, revealing the dependence of antimicrobial activity on zinc concentration (ZID = f(ZnX)). In a few cases, the ZnO NP ZIDs were comparable with the ZIDs of precursory zinc salts (ZnCl_2_, ZnSO_4_, or Zn(OAc)_2_). Since zinc metal presents lower solubility than ZnO (1 μg/L vs. 3.6 μg/L) [149] and much lower solubility than ZnO NPs [150,151,152,153], the metallic zinc in COT–Zn composites presented lower solubility in inoculum media than ZnO NPs and, consequently, a lower ZID. Therefore, the process of releasing antimicrobial zinc ions from COT–Zn (COT–Zn→COT–Zn(OH)_2_→COT + Zn^2+^) is much longer, and these composites should preserve their antimicrobial nature/characteristics for much longer.

### 3.4. Cytotoxicity

Cytotoxicity assays [154,155,156] are crucial in biomaterial science with respect to the therapeutic potential of nanocomposites and nanostructures, as well as the legal and normative requirements for medical devices and biomaterials [157,158,159,160,161,162,163,164]. These include an array of methods, mostly fluorescent and colorimetric, providing quantitative estimations of the number of viable cells in a culture [154,155]. The neutral red uptake (NRU) assay is one of the most used cytotoxicity tests in biomedical and environmental applications [165] and is based on the natural tendency of neutral red dye to incorporate to living cell lysozymes. As cells begin to die (a loss of cell viability), their ability to bind neutral red diminishes (decrease in neutral red uptake), corresponding to colorimetric changes.

Zinc salts have proven to exert a strong biological effect, i.e., an antimicrobial effect toward several bacteria [41], as well as a cytotoxic effect toward various mammalian cell viability [166]. Cytotoxicity studies were used to screen the therapeutic potential of zinc-containing nanostructures [167,168,169,170,171,172,173,174,175]. Zn/ZnO coatings are partially soluble in water [149], releasing Zn^2+^ ions. These, in turn, induce cytotoxicity by increasing the excessive intracellular Zn ion concentration [166,176,177]. In order to screen the therapeutic potential of COT–Zn composites, we investigated their cytotoxic action on Balb/c 3T3 fibroblast cells [178], using the neutral red uptake assay [174,178].

### 3.5. Cytotoxicity Experiments

**Figure 9:**
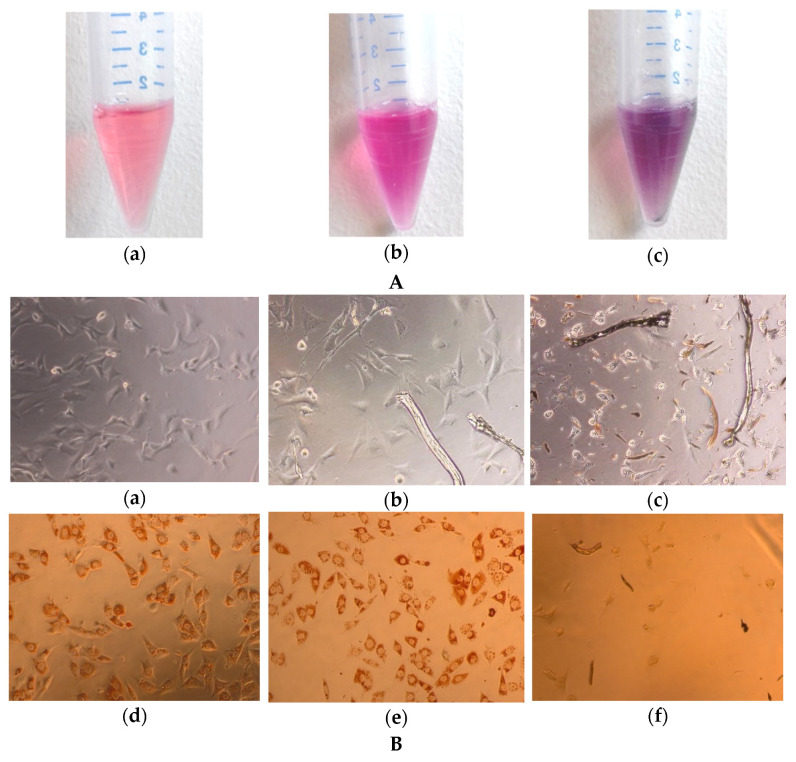
(**A**). Images showing exposure medium treated in the same way as (NC) extracts (**a**), unmodified cotton extract (**b**), and modified cotton extract (**c**), (**B**). Light microscopy images of BALB/3T3 clone A31 cells exposed for 24 h to negative control (**a**,**d**), 100% unmodified cotton extract (**b**,**e**), and 100% extract from COT–Zn sample (**c**,**f**), before and after incubation with NR, respectively.

Macroscopic observations of the extracts showed no changes in transparency for unmodified cotton, while the modified cotton had a visibly darker color, brightening after sedimentation, and leaving a black residue (Figure 9A.). Microscopic analysis of the exposed cells revealed fibers present in all extract samples and small fragments present only in the modified cotton extracts (Figure 9B).

**Figure 10:**
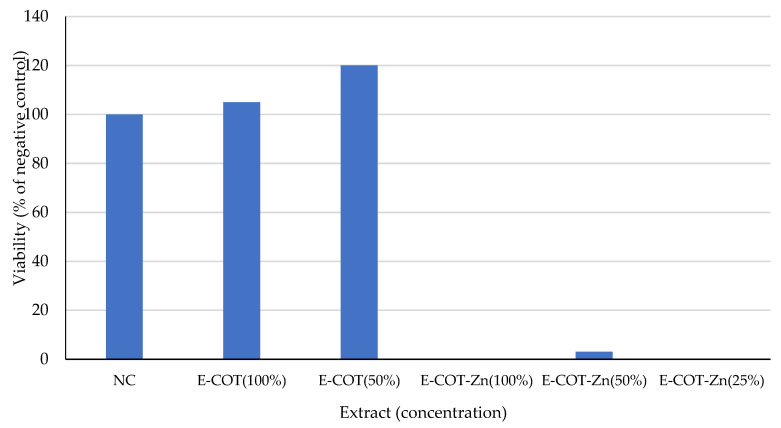
Effect of 24 h exposure of BALB/3T3 clone A31 cells on unmodified and modified cotton extracts, determined with the NRU test. Viability is shown as a percentage of the negative control (NC; exposure medium treated analogously to the extracts).

**Figure 11:**
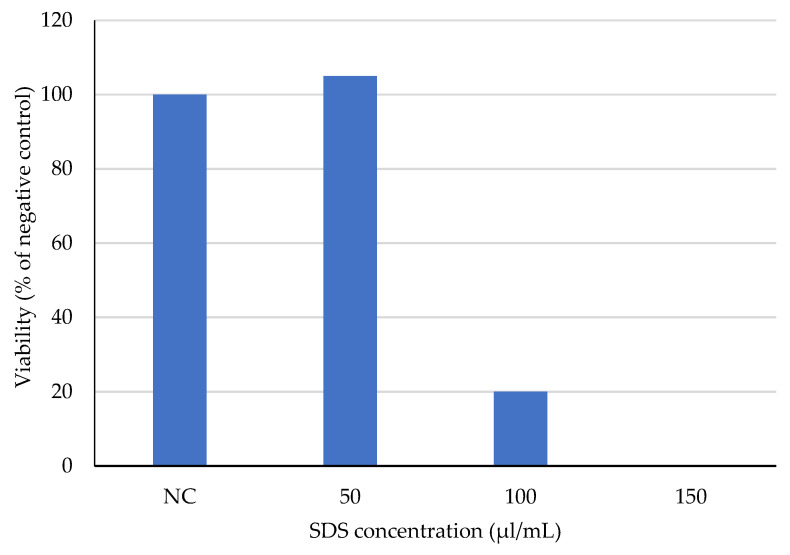
Viability of BALB/3T3 clone A31 cells exposed for 24 h to SDS (positive control), assayed with NRU test. Viability presented as a percentage of the negative control (NC; culture medium with vehicle, i.e., 2% H_2_O in culture medium).

The results of the NRU assay showed no decrease in viability of fibroblasts exposed to the unmodified cotton extracts at both tested concentrations. COT–Zn extracts significantly reduced cell viability, causing almost 100% cell death, irrespective of the extract concentration (Figure 10). Treatment of cells with SDS resulted in a concentration-dependent decrease in cell viability (Figure 11).

Zinc compounds also reveal strong anticancer activity (e.g. [136,179,180,181]) reflected additionally by nearly 2400 document results on Anticancer Zinc [182] and 1–700 documents results on Anticancer Activity of Zinc abstracted in the Scopus Base [183]. Therefore the COT-Zn composites with cytotoxic activity against BALB/3T3 clone A31 cells should also reveal anticancer character. These investigations will be continued.

## 4. Conclusions

In summary, COT–Zn composites with different Zn content were prepared by DC magnetron sputtering technology using a zinc metal target. The composite samples were characterized by SEM, EDS, and FAAS. The biological properties of the materials were verified by cytotoxicity screening and antimicrobial activity tests against colonies of *E. coli* and *S. aureus* bacteria and *A. niger* and *C. globosum* fungi. The in vitro determined antimicrobial properties of COT–Zn composites revealed the antibacterial and antifungal activities. In vitro studies showed also that COT–Zn composites containing merely 9 g/kg Zn were cytotoxic. The ability to adapt clean and zero-waste magnetron sputtering methods to an industrial scale provides the possibility to obtain sustainable materials for use in a variety of applications. The prepared fiber composites have great application potential as an antimicrobial material in the field of biomedical engineering (e.g., rehabilitation, medical devices); however, due to their cytotoxicity they have limited possibilities of use as a material interacting with cells of the human body.

## Figures and Tables

**Table 1:** Representative composite cotton/zinc salts/MNPS.

No	Preparation	Antibacterial Activity	Ref.
2.1	COT–ZnO (Zn^2+^(NaOH)→Zn(OH)_2_→ZnO) ZnO deposition by ‘pad–dry–cure’ method.	COT–ZnO showed excellent antibacterial activity against Kp and Sa, e.g., COT–ZnO (1.6%) exhibited 99.9%rv after 24 h exposure. Application onto cotton fabrics to impart antibacterial and UV protection function.	[62]
2.2	COT–ZnO (Zn^2+^(NH_3_ˣH_2_O)→Zn(OH)_2_→ZnO) ZnO deposition by ultrasound irradiation.	COT–ZnO showed excellent antibacterial activity against Kp and Sa, e.g., COT–ZnO (0.8%) exhibited 99.9%rv after 2 h exposure. Potential application as coated bandages.	[63]
2.3	COT–CTS (0.3%)–ZnO (0.2–2 mM) ZnO one-step sonochemical deposition on COT/CTS	Enhanced antimicrobial effect (Sa (98.5% rv) and Ec (99.9%rv)) after 1 h incubation and high washing stability enable recommending this antibacterial textile for uses in a hospital environment to prevent the spread of nosocomial infection	[64]
2.4	COT–ZnO (0.8%) (COT + ZnO NPs (1 mM)+ C-ase (2%)), ZnO sonochemical coating.	The antibacterial efficiency of COT–ZnO (Ec 67% and Sa 100% after 1 h incubation) resisted the intensive laundry regimes used in hospitals.	[65]
2.5	COT–ZnO	In situ nanoscale synthesis of ZnO on the surface of cotton fabrics (COT + ZnCl_2_ + NaOH→COT–ZnO) led to high antibacterial activity against Ec and Ec.	[66]
2.6	COT–ZnO; COT–PVP–ZnO	The antibacterial efficiency of COT–ZnO after 2 h incubation: COT–ZnO (5 mg/L)—Ec, 81%rv and Sa, 57%rv; COT–PVP–ZnO (5 mg/L)—Ec, 80%rv and Sa, 71%rv; COT–PVP–ZnO (20 mg/L)—Ec, 100%rv and Sa, 100%rv. Potential application as wound cloths, surgical cloths, sportswear and kidswear.	[67]
2.7	COT–R–N^+^Me_3–_ZnO/ZnO	Nano-ZnO films deposited on cotton fabrics (10–16 layers) exhibited excellent antimicrobial activity against Sa.	[68]
2.8	COT–ZnO; COT–BTCA–SiO_2_–ZnO; COT–APTES–BTCA–SiO_2_–ZnO; COT–VTES–SiO_2_–ZnO;	The antibacterial efficiency of hybrids varied for Ec from 96%rv to 99%rv and for Sa from 55%rv to 90%rv; after 20 washing cycles, it varied for Ec from 57%rv to 91%rv and for Sa from 55%rv to 90%rv. Multi-application potential.	[69]
2.9	COT–ZnO (Zn^2+^(HMTETA, H_2_O)→ZnO)	The antibacterial efficiency of hybrids after 24 h exposure varied for Ec from 91%rv to 97%rv and for Sa from 95%rv to 98%rv.	[70]
2.10	COT–ZnO (Zn^2+^(MMA, EtOEtOH)→ZnO) ZnO coating by a spin coater.	The antibacterial activities of the ZnO-coated fabric were investigated (zone inhibition diameter) against Kp, St (36 mm), Ec (19 mm), Bs (17 mm), and St (20 mm) using 48 h exposure time. Comparable with ampicillin and/or gentamycin.	[71]
2.11	COT–ZnO (Zn^2+^(H_2_O, NH_4_Cl, NH_3_)→ZnO) ZnO synthesized on the surface of COT via a simple wet chemical route.	Antibacterial tests against Sa and Kp (the presence of a significant inhibition zone of at least 1 mm around the fabric) revealed good bacteriostatic activity. However, the negligible reduction in the number of bacteria proved the lack of bactericidal activity.	[72]
2.12	COT–ZnO (1%) (Zn^2+^ (H_2_O, At)) ZnO resuspended in water (20 ppm) was coated on cotton.	The treatment of the cotton fabrics with ZnO-NPs was carried out at a safe dose (20 ppm). At this dose, ZnO-NP-loaded samples exhibited reasonable antibacterial activity against Sa, Bs, Ec, and Pa.	[73]

Bacteria and fungi: At—Aspergillus terreus AF-1; Bs—Bacillus subtilis; Ca—Candida albicans (fungi); Ec—Escherichia coli; Kp—Klebsiella pneumoniae; Pa—Pseudomonas aeruginosa; Sa—Staphylococcus aureus; St—Salmonella typhimurium; %rv—percentage reduction in viability of bacteria/fungi. Reagents: APTES—(3-aminopropyl) triethoxysilane; BTCA—butyltetracarboxylic acid; VTES—vinyltriethoxysilane; HMTETA—hexamethyltriethylene tetramine; MMA—monomethyl amine, EtOEtOH—ethoxyethanol. Fibers/textiles: COT—cotton, CTS—chitosan.

**Table 2:** Extract abbreviations.

Extracts Abbreviations	Starting Materials				Exposure Medium (NC) ^/a^	
	COT	COT–Zn ^/b^	E-COT (100%)	E-COT–Zn (100%)	Extraction ^/c^	Dilution
E-COT (100%)	0.1 g				1.n_1_ mL	
E-COT (50%)			0.5 mL			0.5 mL
E-COT–Zn (100%)		0.1 g			1.n_2_ mL	
E-COT–Zn (50%)				0.5 mL		0.5 mL
E-COT–Zn (25%)				0.25 mL		0.75 mL

^a/^ cDMEM with a lower (5%) concentration of FBS; ^b/^ COT–Zn-10 (1 s) was used for cytotoxicity assays; ^c/^ 1 mL of the exposure medium + the predetermined additional volume of the exposure medium needed for the maximum soaking of the test material (ni).

**Table 3:** Quantitative content of elements in individual samples based on the EDS test.

Sample	Quantitative Content of Elements (wt.%)		
	C	O	Zn
COT	40.04	59.96	-
COT–Zn-10 (1 s)	3.20	17.70	79.73

**Table 4:** Results of determination of zinc content in COT–Zn composite samples.

Sample Name	Zn Concentration (g/kg)
COT	0.01
COT–Zn-5 (1 s)	9.06
COT–Zn-10 (1 s)	20.19
COT–Zn-10 (2 s)	41.52

The results were measured in triplicate and are presented as mean values with a deviation of approximately ±2%.

**Table 5:** Results of antibacterial activity test of zinc compounds and composites.

Sample	Conc.	Zone Inhibition Diameters (mm) ^/^^a^								Ref.
		Gram-Negative				Gram-Positive				
		Ec	Kp	Pa	Pm	Bs	Ef	Sa	Se	
ZnSO_4_	2 mg/mL	17	14	14	15			28	15	[127]
ZnSO_4_	10 mg/mL	23	26	21	23			38	26	
ZnO NPs	1 mg/mL	16				19		18		[128]
ZnO NPs	1 mg/mL	18						15		[129]
ZnO (ZOE)							3			[130]
ZnCl_2_	6 mg/mL	-	-		-	-		-		[131]
Zn(L^i^)_2_(W)_2_	6 mg/mL	-	-		-	10		12		
Zn(L^i^)_2_(L^2^)(W)_2_	6 mg/mL	14	11		14	12		15		
ZnO NPs	50 μg/mL	24	16	26		24		22		[132]
COT ^/b^	0.01 mg/g	0						0		This work ^/c^
COT–Zn-5 (1 s) ^/b^	9.0 mg/g	1						0		
COT–Zn-10 (1 s) ^/b^	20 mg/g	1						1		
COT–Zn-10 (2 s) ^/b^	42 mg/g	1						1		

^/a^ Zone inhibition diameter (ZID), rounded to whole numbers (mm); NPs—nanoparticles; L—ligand (L1= ibup (ibuprofen); L2 = 2′-bipy (2,2′-bipyridine); W = water); ZOE—ZnO–eugenol. ^/b^ Concentration of inoculum: *E. coli*: CFU/mL = 1.5 × 10^8^, *S. aureus*: CFU/mL = 2.5 × 10^8^. ^/c^ ZID determined according to PN-EN ISO 20,645:2006 standard [116]. Bacteria: Bs—*Bacillus subtilis*; Ec—*E. coli*; Ef—*Enterococcus faecalis*; Kp—*Klebsiella pneumoniae*; Pa—*Pseudomonas aeruginosa*; Pm—*Proteus mirabilis*; Sa—*Staphylococcus aureus*; Se—*Staphylococcus epidermis*.

**Table 6:** Results of the antifungal activity test and growth inhibition effects of zinc compounds and composites.

No	Zinc Compounds/Composites ^/a^	Deposition ^/b^	Fungal Average Zone Inhibition Diameters (ZID: mm) ^/c,d^					Ref.
			An	Afl	Afu	Ca	Cg	
1	ZnO	1 mg/mL	8	8				[133]
2	ZnO NPs	1 mg/mL	18			20		[134]
		0.4 mg/mL	35					[135]
		50 μg/mL	16			19		[132]
	ZnO NPs	1 μg/mL	24			28		[136]
	Zn(OAc)_2_	1 μg/mL	20			21		
	ZnO NPs	1 mg/disc	6	8	6			[137]
		10 mg/disc	8	11	8			
		0.02 mg/mL	10	5	7	14		[138]
		0.1 mg/mL	13	10	11	19		
		^/^^e^	0–9 ^/e,f^			0–8 ^/e^		[139]
3	ZnO/CuO (1:1) NPs	0.5 mg/mL	0	0				[140]
4	hAp	5μg	12			13		[141]
	hAp–Zn (15%)	5μg	12			18		
5	Cell/Cts/ZnO	0.25%	9–11					[142]
6	CTS–ZnO film	1 mg/mL	14			4		[143]
7	PS/ZnO-NPs (5%)		19	20				[144]
8	ZnO NPs–eugenol	26 μg/L	24					[145]
9	PLA–ALG–Zn^2+^	1.2%	1				1	[146]
	PLA–ALG–Zn^2+^	4.0%	1				1	
10	COT–ZnO	^/f^	10					[147]
	COT–ZnO–MnO_2_(1:1)	^/f^	12					
11	COT ^/b^	0.01 mg/g	0 ^/g,h^				0 ^/g,h^	This work
	COT–Zn-5 (1 s) ^/b^	9.0 mg/g	0 ^/g,h^				0 ^/g,h^	
	COT–Zn-10 (1 s) ^/b^	20 mg/g	1 ^/g,h^				1 ^/g,h^	
	COT–Zn-10 (2 s) ^/b^	42 mg/g	1 ^/g,h^				1 ^/g,h^	

^/a^ Zinc compounds and composites; Cell—cellulose; COT—cotton; Cts—chitosan; hAp—hydroxyapatite; PLA—polylactide; PS—polystyrene; ZnO NPs—zinc oxide nanoparticles. ^/b^ Deposited on discs as originally assigned (ug/mL, mg/mL, mg/disc; % of zinc in the solution or solid sample). ^/c^ Fungi: An—*Aspergillus niger*; Afl—*Aspergillus*; Afu—*Aspergillus fumigates*; Ca—*Candida albicans*; Cg—*Chaetomium globosum*. ^/d^ Zone inhibition diameter (ZID), rounded to whole numbers (mm); ZID was determined according to PN-EN ISO 20,645:2006 standard [117]. ^/e^ Dependent on the green method applied. ^/f^ Dependent on the green method applied. ^f^ Cotton patch (5 × 5 cm^2^) saturated for 5 min in 10% aqueous solution of ZnO and/or MnO_2_/ZnO. ^/g^ Concentration of inoculum: *A. niger*: CFU/mL = 3.5 × 10^6^, *C. globusum*: CFU/mL = 3.0 × 10^6^. ^/h^ Visible growth on sample surface.

## Data Availability

Not applicable.
